# Supplementary material for: Multimodal state-dependent connectivity analysis of arousal and autonomic centers in the brainstem and basal forebrain
Source: Imaging Neurosci (Camb). 2025 Jul 21;3:IMAG.a.91. doi: 10.1162/IMAG.a.91 (PMC12330857; doi:10.1162/IMAG.a.91)
Supplement: Supplementary Material [file IMAG.a.91_supp.pdf]

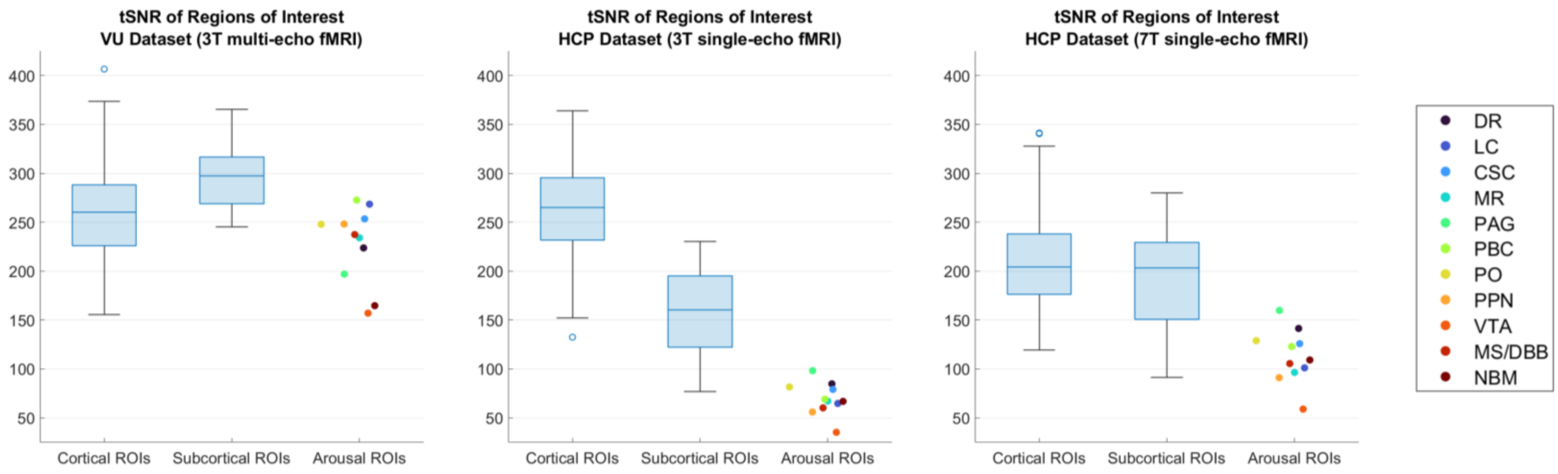

Supplementary Fig. 1. Temporal signal-to-noise ratio (tSNR) of the brain regions-of-interest (ROIs) in the VU 3T-ME, HCP 3T, and HCP 7T datasets. The tSNR is averaged over all the subjects in each fMRI dataset, and the boxplots depict the distribution of the tSNR across the ROIs. The arousal ROIs include 9 brainstem regions from the Harvard Ascending Arousal Network (AAN) atlas Version 1.0 (Edlow et al., 2024; Edlow et al., 2012) and two bilateral basal forebrain regions from the Jubrain Anatomy Toolbox (Zaborszky et al., 2008). The cortical ROIs are defined from the Schaefer atlas (200 ROIs, 17 networks) (Schaefer et al., 2018), and the subcortical ROIs are defined from the Melbourne atlas (32 ROIs) (Tian et al., 2020).

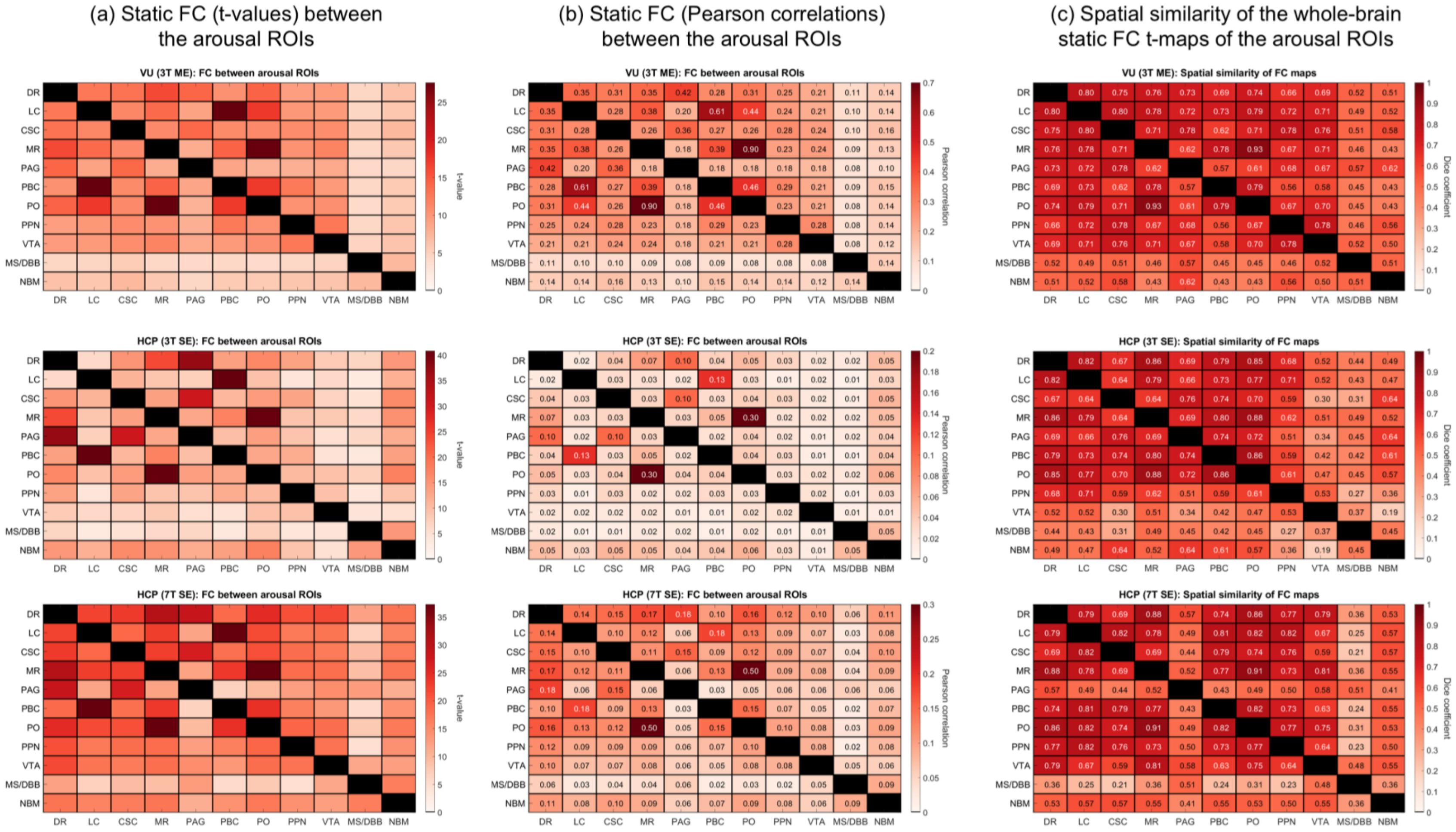

Supplementary Fig. 2. (a-b) Static functional connectivity (FC) of the subcortical arousal regions with each other in the VU 3T-ME, HCP 3T, and HCP 7T datasets for the mCSF/WM preprocessing pipeline. The FC is depicted as the Pearson correlation averaged across all the subjects in each dataset and as t-values derived for the group average correlation in each dataset. (c) Spatial similarity (Dice similarity coefficient) of the whole-brain static FC t-maps of the subcortical arousal ROIs with each other.

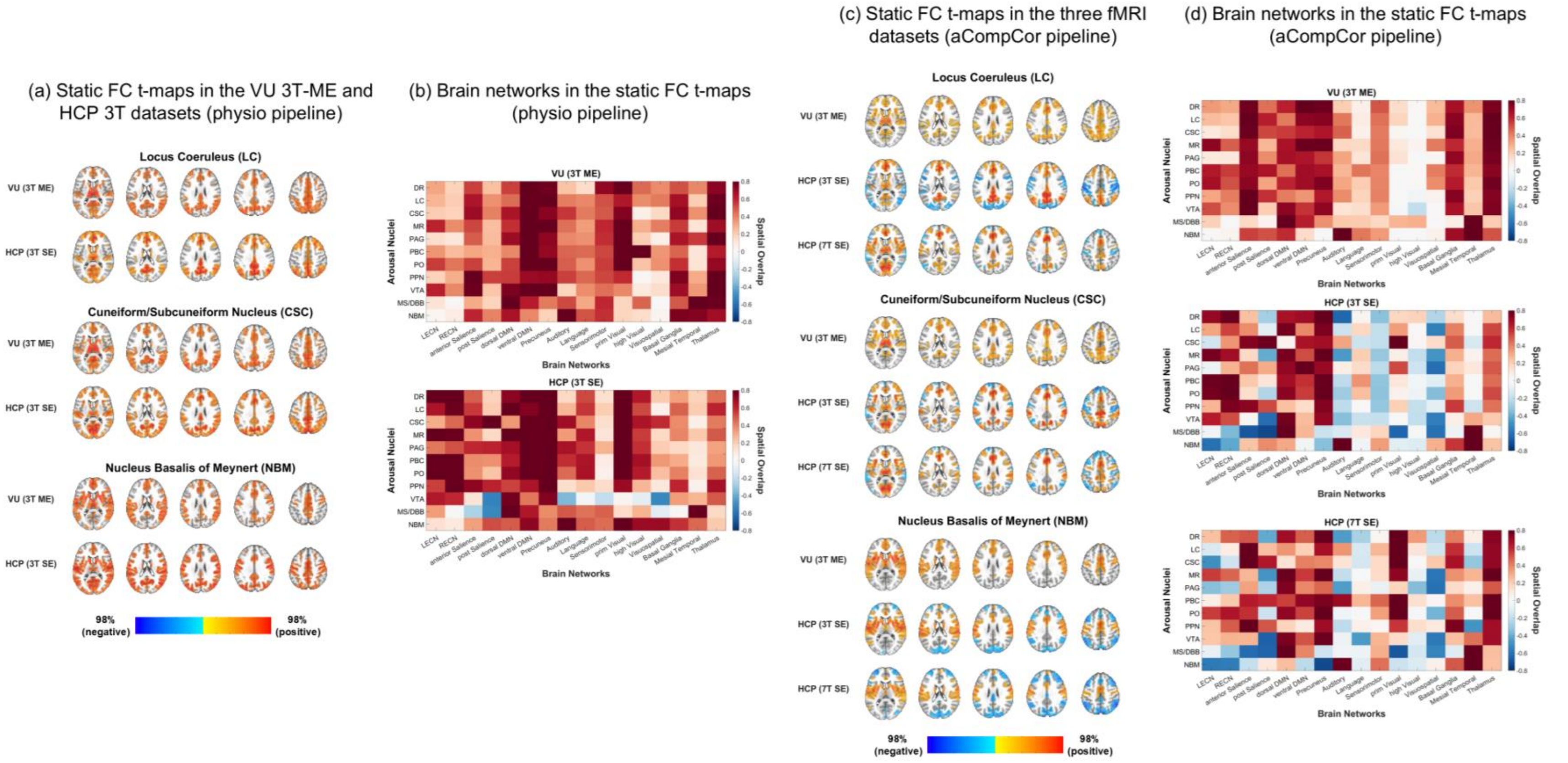

Supplementary Fig. 3. (a, c) Static functional connectivity (FC) t-maps of the locus coresuleus (LC), cuneiform/subcuneiform nucleus (CSC), and nucleus basalis of Meynert (NBM) in the VU 3T-ME, HCP 3T, and HCP 7T datasets for the physio and aCompCor preprocessing pipelines. The FC t-maps were thresholded at 40% of the top t-values in the gray matter and at $p < 0.05$ (voxel-wise false discovery rate [FDR]-corrected over the entire gray matter volume). AFNI was used for visualization of the t-maps (@chauffeur_afni function; upper functional range set to the 98th percentile). (b, d) Spatial overlap of the thresholded static FC t-maps of the subcortical arousal regions with 16 canonical brain network templates from the FINDLAB and Melbourne atlases (Shirer et al., 2012; Tian et al., 2020). A positive value for the spatial overlap corresponds to mostly positive correlations within the brain network template while a negative value corresponds to mostly negative correlations.

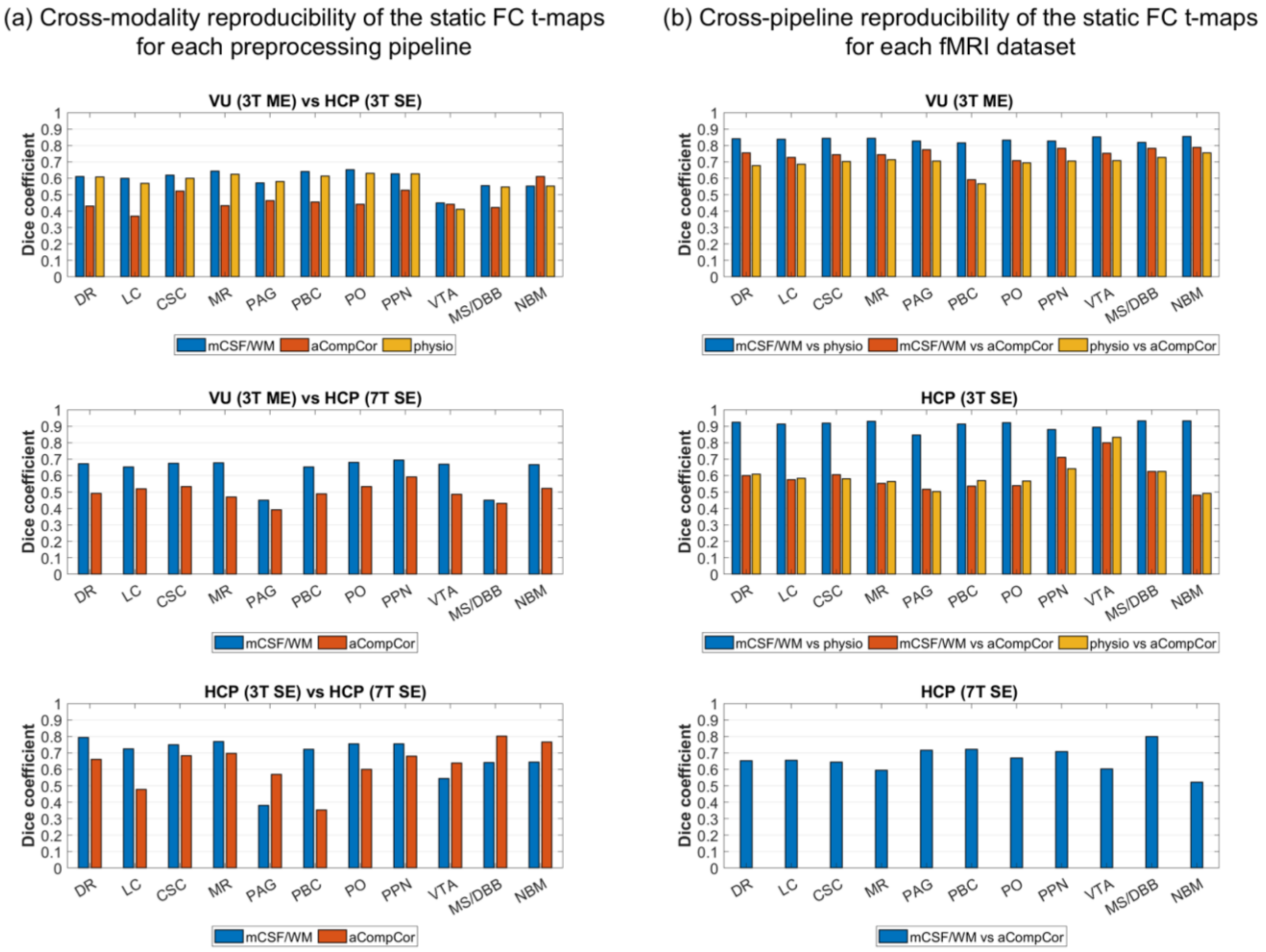

Supplementary Fig. 4. (a) Spatial reproducibility (Dice similarity coefficient) of the thresholded static functional connectivity (FC) t-maps between the VU 3T-ME, HCP 3T, and HCP 7T datasets for each preprocessing pipeline (mCSF/WM, physio, and aCompCor). (b) Spatial reproducibility (Dice similarity coefficient) of the thresholded static FC t-maps between the mCSF/WM, physio, and aCompCor pipelines for each fMRI dataset.

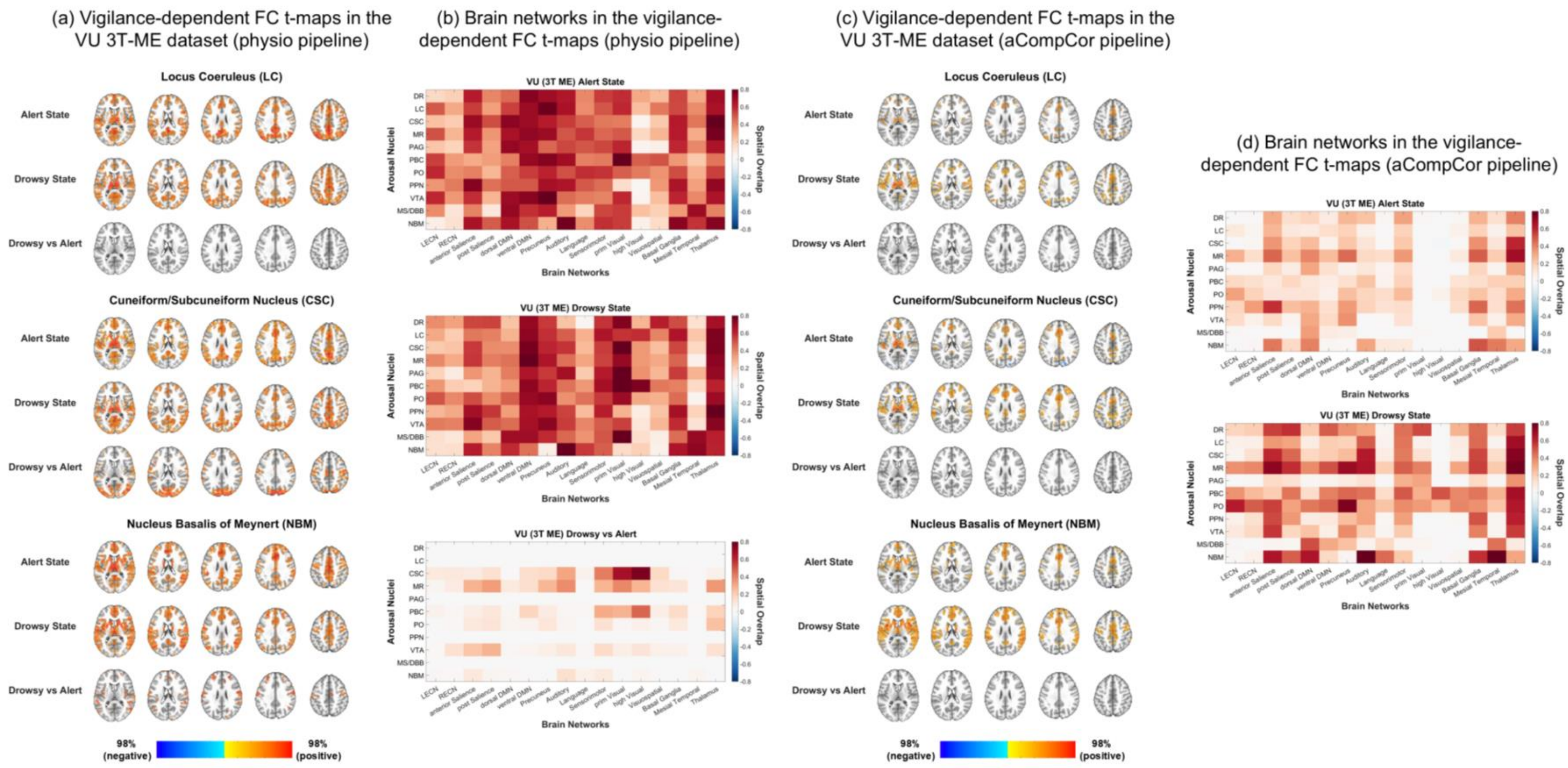

Supplementary Fig. 5. (a, c) Vigilance-dependent functional connectivity (FC) t-maps of the locus coresuleus (LC), cuneiform/subcuneiform nucleus (CSC), and nucleus basalis of Meynert (NBM) in the VU 3T-ME dataset for the physio and aCompCor preprocessing pipelines. The FC t-maps were thresholded at 40% of the top t-values in the gray matter and at $p < 0.05$ (voxel-wise false discovery rate [FDR]-corrected over the entire gray matter volume). AFNI was used for visualization of the t-maps (@chauffeur_afni function; upper functional range set to the 98th percentile). (b, d) Spatial overlap of the thresholded vigilance-dependent FC t-maps of the subcortical arousal regions with 16 canonical brain network templates from the FINDLAB and Melbourne atlases (Shirer et al., 2012; Tian et al., 2020). A positive spatial overlap corresponds to mostly positive correlations within the brain network template for the single-state FC t-maps or to greater FC in drowsy compared to alert for the drowsy versus alert FC t-maps. A negative spatial overlap corresponds to mostly negative correlations or to greater FC in alert compared to drowsy.

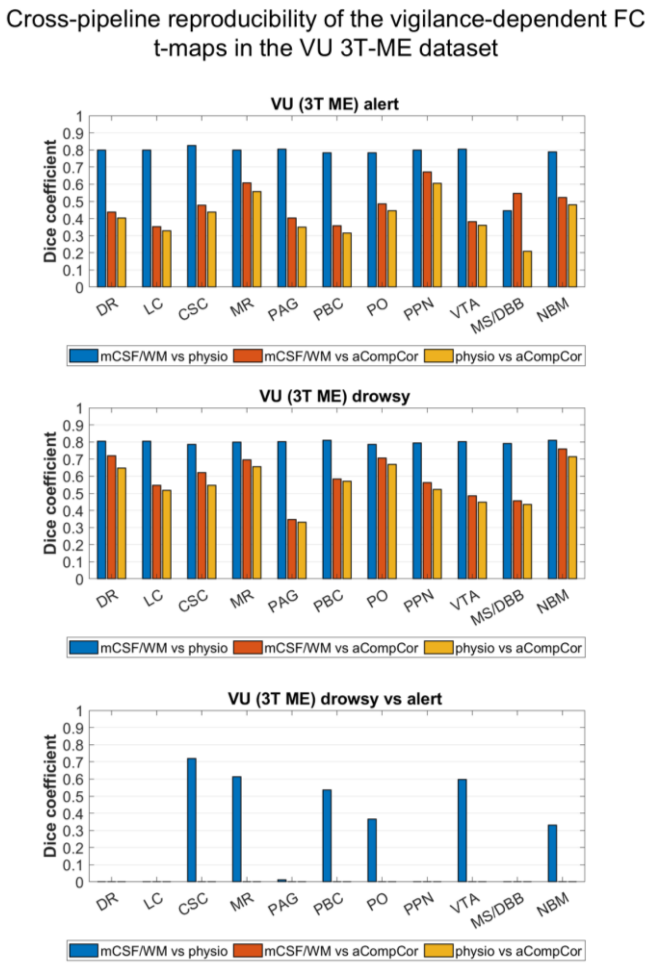

Supplementary Fig. 6. Spatial reproducibility (Dice similarity coefficient) of the thresholded vigilance-dependent functional connectivity (FC) t-maps between the mCSF/WM, physio, and aCompCor pipelines in the VU 3T-ME dataset.

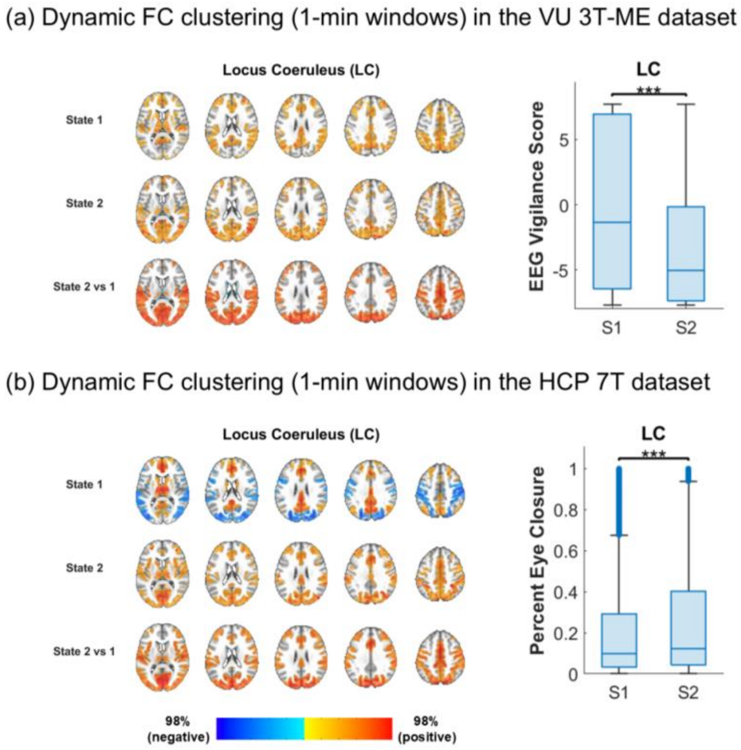

Supplementary Fig. 7. (a-b) State-dependent functional connectivity (FC) t-maps of the locus coreuleus (LC) in the VU 3T-ME and HCP 7T datasets for the mCSF/WM preprocessing pipeline, and comparison of vigilance metrics (i.e., EEG vigilance score in the VU 3T-ME dataset and percent eye closure in the HCP 7T dataset) between the two states. Unsupervised clustering of the dynamic whole-brain correlation patterns (1-min window size; 50% overlap) was used to derive the states. The FC t-maps were thresholded at 40% of the top t-values in the gray matter and at $p < 0.05$ (voxel-wise false discovery rate [FDR]-corrected over the entire gray matter volume). AFNI was used for visualization of the t-maps (@chauffeur_afni function; upper functional range set to the 98th percentile). Asterisks indicate a significant difference of the vigilance metrics between the two states at ***$p < 0.001$.

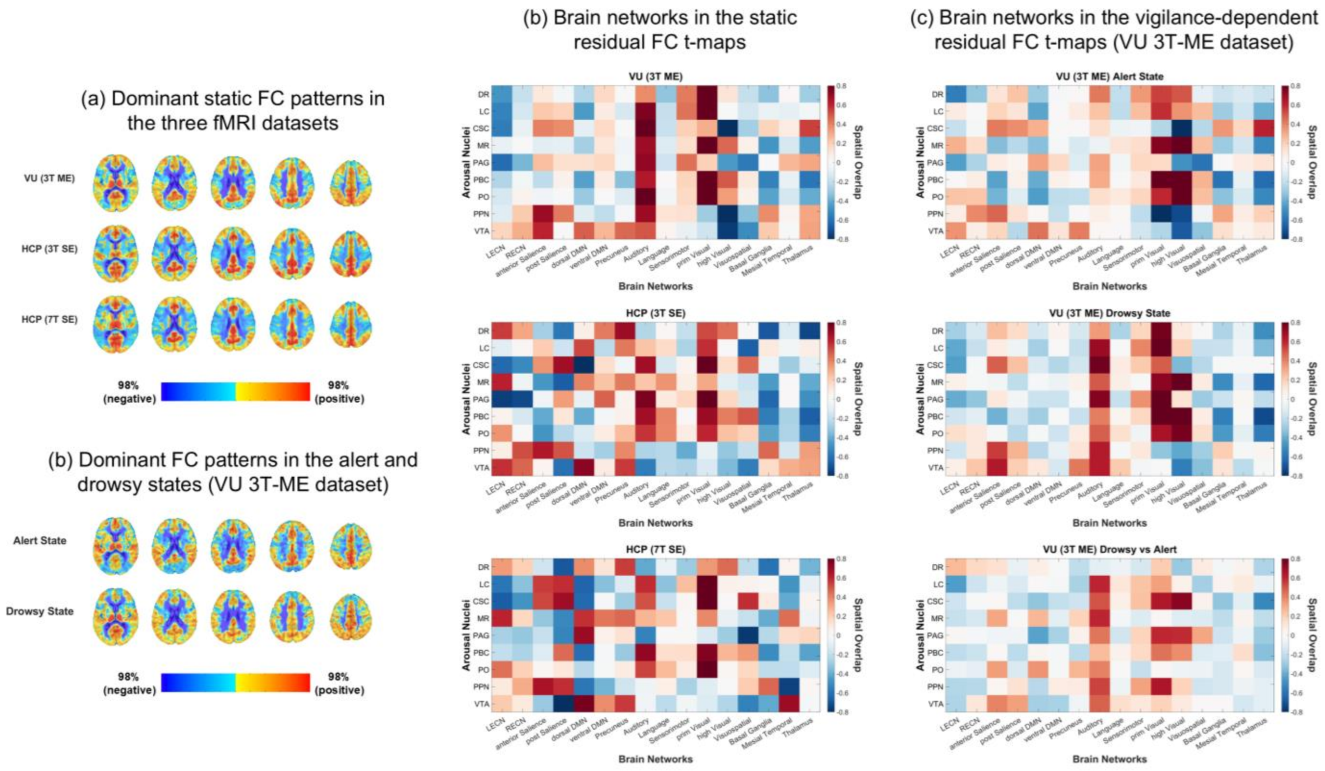

Supplementary Fig. 8. (a) Group-level dominant static functional connectivity (FC) patterns obtained via principal component analysis (PCA) of the static FC t-maps of the brainstem nuclei in the VU 3T-ME, HCP 3T, and HCP 7T datasets (mCSF/WM preprocessing pipeline). (b) Group-level dominant FC patterns in the alert and drowsy states obtained via PCA of the single-state FC t-maps of the brainstem nuclei in the VU 3T-ME dataset (mCSF/WM preprocessing pipeline). (c-d) Spatial overlap of the static and vigilance-dependent residual FC t-maps (thresholded at 40% of the top t-values in the gray matter; no p-value threshold) with 16 canonical brain network templates from the FINDLAB and Melbourne atlases (Shirer et al., 2012; Tian et al., 2020). The static residual FC t-maps were obtained after regressing out the dominant static FC pattern from the static FC maps of each subject. The vigilance-dependent residual FC t-maps were obtained after regressing out the corresponding dominant alert or drowsy FC pattern from the FC maps of each alert and drowsy epoch. None of the brainstem nuclei had significant drowsy versus alert differences in their residual FC maps after voxel-wise false-discovery rate (FDR) correction.

References

Edlow, B. L., Olchanyi, M., Freeman, H. J., Li, J., Maffei, C., Snider, S. B., Zollei, L., Iglesias, J. E., Augustinack, J., Bodien, Y. G., Haynes, R. L., Greve, D. N., Diamond, B. R., Stevens, A., Giacino, J. T., Destrieux, C., van der Kouwe, A., Brown, E. N., Folkerth, R. D., Fischl, B., & Kinney, H. C. (2024, May). Multimodal MRI reveals brainstem connections that sustain wakefulness in human consciousness. *Sci Transl Med, 16*(745), eadj4303. https://doi.org/10.1126/scitranslmed.adj4303

Edlow, B. L., Takahashi, E., Wu, O., Benner, T., Dai, G., Bu, L., Grant, P. E., Greer, D. M., Greenberg, S. M., Kinney, H. C., & Folkerth, R. D. (2012, Jun). Neuroanatomic connectivity of the human ascending arousal system critical to consciousness and its disorders. *J Neuropathol Exp Neurol, 71*(6), 531-546. https://doi.org/10.1097/NEN.0b013e3182588293

Schaefer, A., Kong, R., Gordon, E. M., Laumann, T. O., Zuo, X. N., Holmes, A. J., Eickhoff, S. B., & Yeo, B. T. T. (2018, Sep 1). Local-Global Parcellation of the Human Cerebral Cortex from Intrinsic Functional Connectivity MRI. *Cereb Cortex, 28*(9), 3095-3114. https://doi.org/10.1093/cercor/bhx179

Shirer, W. R., Ryali, S., Rykhlevskaia, E., Menon, V., & Greicius, M. D. (2012, Jan). Decoding subject-driven cognitive states with whole-brain connectivity patterns. *Cereb Cortex, 22*(1), 158-165. https://doi.org/10.1093/cercor/bhr099

Tian, Y., Margulies, D. S., Breakspear, M., & Zalesky, A. (2020, Nov). Topographic organization of the human subcortex unveiled with functional connectivity gradients. *Nat Neurosci, 23*(11), 1421-1432. https://doi.org/10.1038/s41593-020-00711-6

Zaborszky, L., Hoemke, L., Mohlberg, H., Schleicher, A., Amunts, K., & Zilles, K. (2008, Sep 1). Stereotaxic probabilistic maps of the magnocellular cell groups in human basal forebrain. *Neuroimage, 42*(3), 1127-1141. https://doi.org/10.1016/j.neuroimage.2008.05.055
